# Polymer-Based Flame-Retardant Asphalt: A Comprehensive Review of Materials, Performance, and Evaluation Methods

**DOI:** 10.3390/polym17243272

**Published:** 2025-12-09

**Authors:** Maja Jokic, Jiemin Zhang, Imrana I. Kabir

**Affiliations:** School of Mechanical and Manufacturing Engineering, University of New South Wales, Sydney, NSW 2052, Australia

**Keywords:** asphalt, polymer modification, fire retardancy, thermal stability, mechanical performance, sustainable materials

## Abstract

The growing demand for durable, fire-resistant, and sustainable pavements has intensified research on asphalt as a polymeric composite system. This review provides a comprehensive analysis of asphalt from the perspective of polymer science, focusing on (1) material composition: asphalt chemistry and polymer–binder interaction, and the introduction of polymer modifiers; (2) material properties: rheology, thermal stability, mechanical properties and flame retardancy; and (3) evaluation methods: derivative thermogravimetric analysis, cone calorimeter, scanning electron microscope and computer simulation. Applications in road infrastructure, industrial surfaces, and high-temperature environments are discussed, emphasizing how polymer modifications enhance performance under operational stresses. Evaluation methodologies, including wheel-tracking tests and thermogravimetric and derivative thermogravimetric analysis, are critically reviewed to quantify deformation, thermal degradation, and fire-resistance mechanisms at both microstructural and molecular levels. Several key challenges remain, including understanding the long-term interaction between polymers and asphalt, optimizing the dispersion of reinforcing materials, and maximizing the performance of recycled polymers. This review aims to guide future research on polymer-modified asphalt systems to achieve safer, more durable, and more sustainable pavement solutions.

## 1. Introduction

Asphalt is one of the most basic components of modern infrastructure and, like concrete and steel, is an indispensable element in building global transportation networks. It has facilitated the rapid development of highways, airports and urban roads, while supporting economic growth and social mobility [1]. With the acceleration of urbanization, the demand for high-performance pavement systems continues to grow. Modern roads not only have to withstand increasing traffic loads but also environmental pressures such as temperature fluctuations, humidity, and oxidative aging. With increasing urbanization, pavements must endure heavier traffic and environmental stresses, including temperature changes, humidity, and aging. Beyond structural demands, asphalt flammability presents a serious safety concern. Derived from hydrocarbons, asphalt poses fire risks in enclosed spaces such as tunnels and bridge decks, where limited ventilation and high traffic can accelerate fire spread. Past incidents demonstrate that uncontrolled asphalt fires can cause casualties, structural damage, and long-term disruption of transportation networks [1,2].

Accordingly, research efforts have increasingly focused on developing fire retardant asphalt formulations capable of reducing fire hazards while maintaining essential mechanical properties. Literature in this field has been identified through systematic searches in databases including Web of Science, Scopus and Engineering Village. Keywords such as ‘Asphalt’, ‘Polymer modification’, ‘Fire retardancy’, ‘Thermal stability’, ‘Mechanical performance’ and ‘Sustainable materials’ guided the review. Studies published mainly between 2020 and 2025 were prioritised, with earlier foundational work included where necessary.

A major strategy for improving fire resistance involves incorporating flame retardant additives into asphalt binders or mixtures. The chemical composition of asphalt, which includes saturates, aromatics, resins and asphaltenes (SARA), makes it particularly susceptible to ignition and thermal decomposition. At elevated temperatures, asphalt emits volatile compounds that intensify combustion [3]. Numerous studies have shown that mineral-based and polymer-modified additives can enhance thermal stability and reduce flammability. Common mineral flame retardants, including aluminium hydroxide (ATH), magnesium hydroxide (MH), and layered double hydroxides (LDHs), decompose and release water vapor under heating conditions, thereby cooling the material, diluting flammable gases, and forming a protective residue that acts as insulation [4]. Intumescent flame retardants (IFRs) have also attracted attention for their ability to form an expanding, thermally stable char layer during heating. These components work synergistically to inhibit combustion and limit oxygen diffusion [5]. Expandable graphite (EG) is another widely studied additive that is characterized by rapid expansion at high temperatures to form an insulating layer. Ammonium polyphosphate (APP) is often used in combination with EG as an acid source to promote char formation [6,7]. Recent studies have also explored bio-based flame retardants, such as chitosan, alginate, and natural biopolymers, which provide fire resistance while also improving sustainability [8].

Achieving fire resistance without affecting mechanical properties has always been a challenge for asphalt mixtures. Excessive additives can reduce the workability of asphalt mixtures, increase brittleness, alter rheological properties, and reduce the cohesion between aggregates [6]. Fire performance assessment requires specialized testing methods, including cone calorimetry, thermogravimetric analysis (TGA), and smoke density measurement [3,6]. These tests can provide key indicators such as ignition time, heat release rate, mass loss, and smoke toxicity. Advanced simulation methods can further supplement experimental results. Molecular dynamics (MD) models can study the composition, aging, and interactions between additives in asphalt [9,10]. Finite element modelling (FEM) helps analyse heat transfer and material response [11,12]. Computational fluid dynamics (CFD) uses tools such as fire dynamics simulators and ANSYS 2025 R1 to simulate flame propagation and smoke movement in tunnel environments [13,14,15,16], providing insights into fire dynamics and emergency planning [17,18].

Overall, this review synthesises current knowledge on FR asphalt, highlighting the relationships among chemical composition, additive modification, mechanical behaviour, and fire performance. The aim is to clarify critical factors influencing flame retardancy and provide direction for designing high-performance, safe, and sustainable asphalt materials.

## 2. Background

Asphalt binder is a colloidal system composed of SARA. During fire exposure, lighter saturates and aromatics volatilise rapidly and ignite, while the heavier asphaltene fraction contributes to limited char formation [19]. The volatile components produce high HRR and dense smoke, making asphalt inherently flammable. These risks are intensified in enclosed settings such as tunnels, where restricted ventilation accelerates smoke accumulation and reduces visibility [20].

Recent studies have advanced the understanding of asphalt’s thermal decomposition behaviour. Techniques such as TGA coupled with mass spectrometry reveal that asphalt undergoes staged thermal degradation, with the early release of combustible volatile organic compounds (VOCs) responsible for rapid flame spread and high flammability [21]. Although asphaltene-derived char forms a partial thermal barrier, its protective effect is inadequate under severe fire conditions, allowing continued ignition and heat penetration [1,22].

Aggregate type further influences fire performance. Limestone aggregates exhibit mild fire-retardant behaviour because their endothermic decomposition releases water vapour, which cools the mixture and dilutes flammable gases [23]. By contrast, siliceous aggregates provide minimal thermal protection [24]. Combustion of asphalt can release hazardous substances including carbon monoxide (CO), carbon dioxide (CO_2_), polycyclic aromatic hydrocarbons (PAHs), and soot, creating additional health concerns during fire incidents [4,19].

Fire performance assessments rely on regulatory standards such as EN 13501 and ASTM E1354 to determine whether materials meet fire safety standards or can be considered non-combustible [25,26]. These standards provide a framework for testing novel flame-retardant asphalt formulations. Studies on the fire behaviour of asphalt pavements using cone calorimetry [27,28] and TGA offer further insights into the thermal stability and decomposition pathways of binders and flame-retardant additives [19,29]. Complementary techniques such as differential scanning calorimetry (DSC) and escape gas analysis (EGA) provide more details on heat flow, chemical transformations, and toxic gas emissions during combustion [29,30].

CFD simulations have become essential for understanding fire development in confined environments. Tools such as FDS enable simulation of heat release, flame spread, smoke transport, and toxic gas dispersion in tunnel geometries, offering detailed insights into the dynamic progression of fires under restricted ventilation [17,31]. Recent advances in multiphase CFD modelling allow improved prediction of soot formation and gas–particle interactions, strengthening tunnel fire risk assessments [32]. These modelling approaches complement laboratory data and support fire-safety engineering and emergency response planning [26].

Despite notable progress in experimental and computational methods, challenges remain in linking laboratory tests to full-scale fire behaviour. Zhang et al. [31] observed that small-scale tests capture relative trends in combustibility and additive effectiveness but do not reproduce transient fire phenomena such as flame spread dynamics, ventilation interactions, or smoke stratification. Lannon et al. [33] similarly reported significant differences between bench-scale and full-scale fire results, underscoring the limitations of current testing approaches and the need for more representative protocols. Consequently, effective fire risk assessment requires a multi-scale methodology that integrates standardised testing, high-fidelity computational modelling, and validation using real fire case studies [1,17,31]. This combined approach enhances the reliability of FR asphalt formulation and supports the development of safer, more fire-resilient pavement systems.

## 3. Materials

The fire performance of asphalt mixtures depends significantly on the type and concentration of additives incorporated into the binder–aggregate matrix. FR additives function by altering thermal decomposition pathways, promoting char formation, releasing non-flammable gases such as water vapor, and creating physical barriers that limit heat and mass transfer during combustion.

### 3.1. Matrix

The asphalt matrix comprises bituminous binder and mineral aggregates, providing mechanical integrity and load distribution [34]. The binder, primarily composed of SARA, has asphaltenes that impart stiffness and thermal stability, promoting char formation during combustion, while maltenes govern flow and adhesion [35,36]. SARA distribution also affects viscoelasticity, oxidative aging, and long-term pavement durability [37,38,39,40].

Table 1 summarizes the main components of asphalt and their effects on mechanical and fire performance. Bituminous binders provide cohesion, flexibility, and viscoelasticity, with thermal stability influenced by SARA distribution [19,41]. Saturates and aromatics affect binder flow, adhesion, and volatile emissions [42], while resins stabilize asphaltenes, enhancing stiffness and char formation [43,44]. Asphaltenes improve stiffness and refractoriness [45,46]. Mineral aggregates form the structural skeleton, and certain types, such as limestone, offer additional flame retardancy via endothermic decomposition [47,48,49]. Polymer modifiers, including SBS and rubber powder, enhance elasticity, fatigue resistance, and thermal stability but may increase VOC emissions [50,51,52,53]. Optimizing these interactions is key to achieving both mechanical durability and fire safety.

### 3.2. Additives

Incorporating FRs into asphalt enhances pavement safety and sustainability. Conventional asphalt is highly flammable, especially in enclosed spaces such as tunnels and airports [60,61]. FR additives reduce fire risk while improving mixture stability [62,63]. Polymer-modified, eco-friendly additives enhance flame resistance [64]. Xu et al. [6] reported that mixtures containing ATH, MH, expanded graphite, and coated red phosphorus reduced smoke and peak heat release while increasing residual mass by 20%, and Sheng et al. [8] confirmed the effectiveness of halogen-free FR systems in tunnels.

FR additives also enable reduced reliance on hydrocarbon binders and support bio-based or nanoscale materials for sustainability [65,66]. Nano clay, graphene derivatives, and nano silica reduce flammability and enhance stiffness and fatigue resistance [66,67], while bio-phosphates improve viscosity and anti-aging properties [65]. Synergistic systems, including ATH, MH, and LDHs, are highly effective [68,69,70]. Polymer and intumescent systems, including nano-enabled FRs, form insulating foamed char layers [71], and chitosan, alginate, and lignin-based nanomaterials reduce flammability while improving stiffness and durability [72].

- Expandable Graphite

EG is a highly efficient halogen-free flame retardant suitable for asphalt binders, primarily due to its excellent expansion properties [73]. When heated, EG expands to form a protective char layer, limiting oxygen diffusion, reducing heat transfer, and slowing the decomposition of the binder, thereby inhibiting flame spread in high-risk environments such as tunnels [74]. Compared to halogenated flame retardants, EG produces less toxic fumes and offers better environmental and safety performance [75]. It is compatible with most asphalt binders and can be used in combination with additives such as APP to enhance char formation and thermal stability [76,77]. Synergistic effects with mineral hydroxides or nano clays can result in endothermic, gas dilution, and barrier layer formation [67]. Studies of nano-expanded graphite (nano-EG) and surface-modified expanded graphite have shown improved dispersibility, specific surface area, and char formation efficiency, while having a smaller impact on the rheological properties of binders [71,78]. Overall, expanded graphite is a highly efficient and environmentally friendly material for preparing refractory asphalt, particularly suitable for closed structures with high requirements for fire safety and structural stability.

- Ammonium Polyphosphate

APP primarily functions as an acid source within intumescent FR systems for asphalt binders. Lim et al. [79] showed that APP promotes the dehydration of organic components in the binder and catalyses the formation of a dense, phosphorus-rich char layer. This char acts as an effective protective barrier, shielding the underlying asphalt from heat and oxygen exposure. At the microstructural level, Liang et al. [80] demonstrate that APP facilitates crosslinking of binder constituents during thermal exposure, resulting in a cohesive char layer that resists erosion and thermal degradation. Such characteristics make APP a critical component in formulations aimed at enhancing the thermal stability and fire resistance of asphalt composites. Additionally, APP’s interaction with other flame retardants improves char quality and thermal shielding, which further supports its use in multi-additive asphalt systems [81].

- Aluminium Hydroxide

ATH is one of the most commonly used mineral-based FR additives in asphalt due to its excellent thermal stability and multiple flame suppression mechanisms. Yang et al. [82] showed that ATH undergoes endothermic decomposition, absorbing significant heat from the asphalt matrix, which lowers the temperature and delays ignition. During decomposition, Li et al. [3] demonstrate that ATH releases water vapor, which dilutes flammable pyrolysis gases and reduces oxygen concentration in the combustion zone, effectively limiting fire spread. Wei et al. [7] showed that the residual aluminium oxide forms a ceramic-like protective layer on the asphalt surface, acting as both a thermal and physical barrier that reduces heat penetration and volatile release. Studies by Zhu et al. [83] confirmed that ATH-modified asphalt mixtures exhibit lower flame spread rates and peak heat release rates, as well as reduced smoke density and toxic emissions, improving fire safety. However, Zhang et al. [84] noted that dosage control is essential since excessive ATH can compromise the mechanical flexibility and toughness of asphalt. Research by Bonati et al. [85] and Iskender et al. [86] emphasized the need to optimize particle sizes of additives and synergistic combinations with polymers or nano clays to balance fire resistance and mechanical integrity in asphalt pavement applications.

- Magnesium Hydroxide

MH is recognized for its multifunctional FR properties in asphalt binders, offering both enhanced fire resistance and thermal stability. Xu et al. [87] investigated that MH decomposes endothermically, absorbing heat and lowering the surface temperature of asphalt, which delays ignition. It is demonstrated that MH releases water vapor during decomposition, diluting pyrolysis gases and lowering oxygen concentration, thereby reducing fire intensity [88]. At the microstructural level, the magnesium oxide residue forms a thermally stable, ceramic-like protective layer on the asphalt surface, acting as a barrier to heat and oxygen penetration [89]. This layer improves cohesion within the asphalt–binder matrix and strengthens char integrity, slowing fire spread and maintaining pavement stability under high temperatures. Tang et al. [90] also report that MH has minimal adverse effects on the binder’s rheological and viscoelastic properties at optimized dosages. Recent studies by Knight et al. [91] highlighted synergistic effects when MH is combined with advanced nanomaterials like carbon nanotubes (CNTs), reinforcing the char layer and enhancing thermal shielding. This multi-layered FR mechanism combining heat absorption, gas dilution, barrier formation, and microstructure enhancement provides comprehensive fire protection for asphalt pavements in high-risk environments such as tunnels.

- Calcium Hydroxide

Calcium hydroxide acts as an effective flame retardant in asphalt by releasing water during thermal decomposition, which cools the material and dilutes combustible gases. Danny et al. [92] showed that calcium hydroxide reacts with acidic species generated during binder combustion, promoting the formation of a more stable and cohesive char structure. The alkaline nature of calcium hydroxide neutralizes bituminous acids produced under high-temperature conditions, thereby improving the binder’s aging resistance and overall durability [93]. These combined mechanisms not only enhance fire retardancy but also contribute to the longevity and performance stability of asphalt pavements subjected to thermal stress, making calcium hydroxide a valuable additive in FR asphalt formulations.

- Polymer Modifiers

Polymeric modifiers are widely used to enhance both the mechanical and fire-resistant properties of asphalt binders [66]. By increasing elasticity and high-temperature stiffness, polymers improve durability and pavement performance. For instance, SBS-modified asphalt exhibits higher softening points, increased complex modulus, and superior rutting resistance, making it suitable for high-temperature applications [53]. From a fire-safety perspective, improved rheology helps maintain binder cohesion at elevated temperatures, reducing mass loss, flame spread, and delaying ignition [51,94]. Certain polymers also promote char formation during pyrolysis, which acts as a thermal shield, slowing heat transfer, suppressing volatile release, and lowering heat release rates [44,95]. Polymer networks can additionally trap volatile organic compounds, reducing smoke generation during combustion [52].

Synergistic effects are observed when polymers are combined with mineral flame retardants. Elastomer phases enhance toughness, while mineral FRs improve thermal stability and flame retardancy [96,97]. APP and Mg(OH)_2_ together form insulating layers that significantly reduce heat release rates and provide thermal shielding [77]. Key factors influencing performance include polymer type (e.g., SBS, F-EPR, polyethylene), dosage, molecular weight, and compatibility with the binder, affecting softening point, complex modulus, and tensile recovery [52,66,98]. Fire-safety evaluations consider char yield, HRR, ignition delay, and smoke production, with uniform dispersion of polymer and FR phases maximizing both mechanical and fire-retardant performance [68].

- Bio-Based Additives

Bio-based additives have emerged as sustainable FR modifiers for asphalt, providing environmental benefits alongside improved fire performance [99,100]. Natural materials such as lignin, chitosan, alginates, and tannins can partially replace petroleum-derived components while enhancing thermal stability [101]. Their FR mechanism primarily relies on char promotion, as oxygenated functional groups dehydrate upon heating to form stable carbonaceous layers. These layers act as thermal barriers, reducing heat penetration and slowing volatilization of light hydrocarbons, which improves ignition resistance [100,102]. Bio-additives also enhance binder–aggregate adhesion, strengthening cohesion under thermal stress and reducing the risk of spalling or delamination [103].

Beyond thermal protection, bio-based additives moderate combustion, reducing toxic gas and smoke emissions by promoting more complete oxidation of volatiles [104,105]. Lignin-modified asphalt, for example, can increase char yield by up to 20% and delay ignition in cone calorimeter tests [106]. Chitosan and alginate additives improve binder cohesion, moisture stability, and fatigue resistance, contributing to durability under wet and high-temperature conditions [107,108]. These additives align with circular economy principles by valorising agricultural and forestry by-products, reducing waste and the carbon footprint of asphalt production [109].

When combined with mineral fillers such as ATH or MH, bio-based additives form hybrid systems that synergistically enhance fire resistance and mechanical performance [61,68,105,109]. Key factors affecting their effectiveness include chemical composition, thermal decomposition temperature, char yield, and binder compatibility [110,111]. The quality of additive dispersion, particle size, and surface functionalization critically influences char cohesion and binder–aggregate adhesion, essential for mitigating rutting and delamination in fire-prone environments [66,112,113].

- Fibres and Nanoparticles Reinforcement

Advanced reinforcing materials, including fibres and nanoparticles, offer innovative solutions for improving the fire safety and mechanical durability of asphalt pavements [114,115]. Fibres can enhance tensile strength, heat resistance, and structural stability. Basalt fibres exhibit excellent thermal stability and mechanical strength [116,117]. Carbon fibres can improve moisture resistance and cohesion [118]. Cellulose fibres can stabilize binders while reducing bleeding and porosity [119,120]. Functionalized fibres, such as polypropylene grafted fibres, can improve compatibility with binders, ensure uniform dispersion, and enhance mechanical and flame-retardant properties [121,122,123].

Nanoparticles act at the molecular scale, altering the microstructure of binders. Nano clay forms tortuous pathways, slowing the diffusion of oxygen and heat [85]. Nano silica fills micropores, promoting the formation of cohesive char layers [124]. Carbon nanotubes (CNTs) form networks, enhancing char layer structure and limiting cracking during combustion [125,126]. Magnesium hydroxide nanoparticles act as an endothermic flame retardant, releasing water vapor to dilute combustible gases and forming a protective magnesium oxide layer [91].

### 3.3. FR Mechanism

Mineral additives such as EG, APP, and hydroxides (ATH, MH, CaOH_2_) primarily function through mechanisms including endothermic decomposition, water vapor release, and intumescent char formation, which collectively provide thermal stability, heat absorption, and smoke suppression. Bio-based additives leverage natural polymers like cellulose and lignin to increase char yield and fire resistance while promoting environmental sustainability. However, their compatibility with asphalt binders requires careful management.

Polymer modifiers contribute to improved fire resistance and mechanical durability by modifying binder properties and fostering cohesive char layers, though they can introduce risks related to thermal degradation. Polymer modifiers, such as SBS, EVA, and EVA-grafted polymers, play a crucial role in improving the fire resistance of asphalt by altering its physical structure and thermal behaviour. These polymers typically have decomposition temperatures above 300 °C, which allows them to remain stable in the early stages of asphalt heating, thus contributing to improved fire resistance. Mechanistically, polymer modification primarily enhances flame retardancy through rheological properties, char layer cohesion, and heat resistance. Polymers improve the high-temperature rheological properties of asphalt, increasing its softening point and thus reducing its heat flux under heat exposure. This stability delays deformation and slows the early stages of thermal degradation. Many polymer systems promote the formation of a more cohesive and mechanically stable char layer that blocks the intrusion of combustible gases and heat transfer. The addition of reinforcing polymers enhances the overall mechanical durability of asphalt, helping to better maintain structural integrity at high temperatures. This indirectly improves flame retardancy and reduces crack formation. Despite these advantages, polymer modifiers may experience thermal degradation at high temperatures, increase material costs, and complicate processing. These factors must be considered when selecting polymers for practical flame-retardant asphalt formulations.

**Figure 1:**
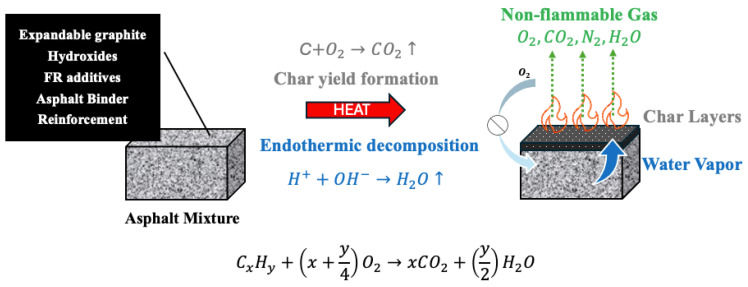
FR mechanism to protect asphalt mixture.

Fibres such as basalt and glass offer physical reinforcement that stabilizes char layers and enhances mechanical strength, thereby slowing fire propagation. Nanoparticles, including carbon nanotubes and nano-clays, deliver advanced barrier effects and radical scavenging capabilities that significantly bolster char integrity and reduce smoke emissions, albeit with challenges related to higher costs and dispersion. Synergistic combinations, such as MH with LDH, enable multi-stage FR mechanisms that optimize fire protection while maintaining pavement performance as shown in Figure 1. Ultimately, balancing fire safety, mechanical properties, and economic feasibility remains essential for developing next-generation asphalt composites tailored for high-risk infrastructure applications such as tunnels and enclosed roadways.

At elevated temperatures, asphalt binder components undergo thermal decomposition, producing volatile gases such as CO, CO_2_, and water vapor, alongside residual char layers. Flame retardants promote endothermic reactions that release water vapor, which absorbs heat and dilutes flammable gases, while simultaneously facilitating the formation of stable char layers that act as physical barriers, limiting oxygen diffusion and heat transfer to the underlying asphalt mixture. These combined chemical and physical effects slow combustion and enhance fire resistance. The schematic in Figure 1, drawn based on literature reports, highlights these processes and the role of flame retardants in modifying decomposition reactions and reinforcing protective char formation.

The combined use of fibres and nanoparticles produces synergistic effects, enhancing asphalt performance at both macro and micro scales [66,127,128]. Fibres provide structural reinforcement to inhibit crack propagation, while nanoparticles block heat transfer, oxygen ingress, and volatile emissions. Hybrid systems, such as basalt fibres with nano-clays or CNTs, have been shown to significantly reduce HRR, smoke density, and improve mechanical strength [129,130]. Key factors influencing performance include additive type, dosage, dispersion quality, particle size, and compatibility with base bitumen [66,131].

Table 2 provides a consolidated comparison of the major FR additives used in asphalt systems, including mineral compounds, bio-based materials, polymer modifiers, fibres, and nanoparticles. As shown in the table, each category exhibits distinct advantages and limitations depending on the intended application, dosage, and compatibility with asphalt binders. Collectively, the literature indicates a clear trend toward more sustainable and multifunctional FR strategies. Rather than treating FR asphalt as a single-purpose FR material, recent studies increasingly frame it as an integrated engineering solution that balances fire performance, mechanical durability, and environmental considerations. This shift reflects growing industry interest in adopting advanced FR systems that not only enhance fire safety but also contribute to the resilience and sustainability of modern road infrastructure.

### 3.4. Asphalt Properties and Their Role in Flame Retardancy

#### 3.4.1. Mechanical Performance and Flame-Retardant Effects

Conventional asphalt mixtures, such as asphalt concrete and mastic asphalt, derive their strength primarily from the binder and aggregate interaction, which governs shear resistance and durability under service loads. Classical mechanical tests such as penetration, softening point, and ductility remain essential for evaluating binder consistency, thermal susceptibility, and fracture tolerance [148,149]. For instance, paving grade binders typically exhibit penetration values of 40 to 100 dmm at 25 °C, softening points around 45 to 55 °C, and ductility greater than 100 cm [150,151]. These parameters indicate that while conventional asphalt offers flexibility and strength, it is vulnerable to rutting at high temperatures and cracking under cold conditions. Flame retardant additives alter these mechanical responses in several ways. Acting as viscosity modifiers or surfactants, they enhance binder and aggregate adhesion and coating uniformity, which not only strengthens rutting resistance but also improves long term structural stability [152]. However, excessive stiffening can reduce ductility, making pavements more susceptible to brittle fracture, an effect particularly critical in cold climates where oxidative hardening already limits flexibility [153,154]. Thus, mechanical tests provide the baseline against which the performance trade-offs of FR modification can be judged.

#### 3.4.2. Rheological Behaviour in Modified Asphalt Systems

Asphalt exhibits viscoelasticity, with elastic (solid like) and viscous (liquid like) behaviour depending on temperature and loading rate [155]. This duality dictates pavement performance: rutting predominates under slow, high temperature loading, while fatigue and cracking emerge under rapid, low temperature stress [156]. Rheological tests such as Dynamic Shear Rheometry (DSR), which measure the complex modulus (|G*|) and phase angle (δ), quantify this balance. A lower δ reflects more elastic recovery, while a higher δ indicates viscous flow [157]. FR systems including intumescent formulations modify these rheological properties by introducing polymeric or resinous additives that interact with asphaltenes and maltenes [158]. These typically enhance rutting resistance by increasing high temperature stiffness but may reduce fatigue life at intermediate temperatures. Nanomaterials such as graphene oxide, carbon nanotubes, or LDHs provide multifunctional benefits, simultaneously improving elasticity, storage modulus, and barrier effects while also delaying ignition [159]. Importantly, FR additives that excessively increase viscosity may hinder mix workability during construction, whereas softening may compromise rutting resistance. Thus, rheological characterization is crucial for optimizing FR formulations without sacrificing structural performance.

#### 3.4.3. Thermal Characteristics and Combustion Resistance

The chemical composition of asphalt, primarily SARA, determines its thermal and flammability [35,48,160]. The binder content varies depending on the petroleum source and refining process, with higher levels of asphaltenes and saturated hydrocarbons, and lower levels of resins and aromatics. Polarity distribution affects colloidal stability, mechanical integrity, and fire sensitivity. Asphaltenes form a dispersed phase in maltene media, increasing stiffness and influencing flame spread [161,162].

**Figure 2:**
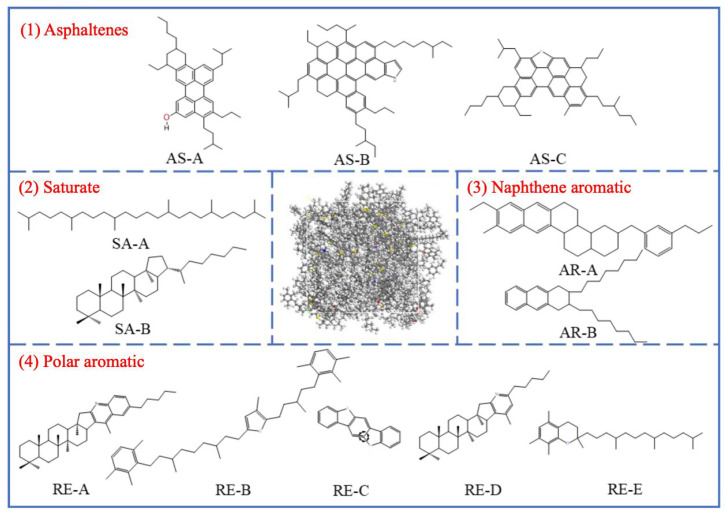
The 12-component model of virgin bitumen. Adapted from [162], MDPI, 2023.

Figure 2 shows a typical molecular structure of asphalt. The most significant chemical characteristic of asphalt is its heterogeneity; therefore, all molecules must be considered for a true chemical description of asphalt [162]. The polarity balance within the colloidal system determines the stability of the binder: asphaltenes form a dispersed phase in maltene media, imparting stiffness to the binder and influencing flame spread. Asphalt itself is flammable because it is primarily composed of hydrocarbons (90% to 95%) and contains heteroatoms such as sulfur, nitrogen, and oxygen, as well as trace amounts of metals (nickel, vanadium, iron) [163].

Table 3 briefly summarizes how each property affects the performance of the pavement under fire conditions and illustrates the benefits and potential drawbacks of adding flame retardants. The presence of heteroatoms accelerates oxidative crosslinking during aging, leading to increased stiffness and brittleness of asphalt. Ultraviolet radiation and high temperatures exacerbate this oxidation reaction, generating carbonyl and sulfoxide groups, thereby reducing the flexibility of asphalt and promoting cracking [164]. Intumescent flame retardant systems slow down the decomposition of hydrocarbon-rich SARA components when heated [165]. By linking molecular structure and polarity distribution to flame retardancy, the inherent thermal vulnerability of asphalt stems from its chemical composition, and effective flame-retardant strategies must address both oxidative aging and hydrocarbon flammability.

## 4. Application of Asphalt

### 4.1. Overview

Based on the material properties discussed in the previous section, the practical value of asphalt is most evident in its wide range of engineering applications. As one of the most commonly used building materials in modern infrastructure, asphalt is widely used in civil, industrial, and emerging sustainable development projects due to its durability, cost-effectiveness, and ability to meet various performance requirements [166]. The primary use of asphalt remains in transportation infrastructure [167]. Each application places different mechanical and environmental requirements on asphalt mixtures; therefore, the selection of binder type, modifier content, and mixture design is crucial for achieving long-term performance. Accordingly, recent research has highlighted the importance of designing asphalt systems specifically tailored to local traffic conditions, climate change, and sustainable development goals [168,169]. These application-driven demands also underscore the importance of reliable evaluation methods, as the suitability of specific asphalt formulations (especially those modified with polymers or flame retardants) must be verified through standardized mechanical, thermal, and fire-resistant performance tests.

- Building and Protective Applications

Beyond road infrastructure, asphalt plays a critical role in building construction as a waterproof and protective material. Asphalt is used in roofing shingles, roll roofing, built-up roofing membranes, and waterproof coatings, offering durable and cost-effective protection against water penetration [170]. Pasetto et al. demonstrated that recycled asphalt shingles for transport infrastructure promote economic sustainability in variable climates [171]. This expands the functional value of asphalt beyond infrastructure into durable building protection.

- Recycling and Sustainability

Asphalt’s recyclability is a core strength in sustainable construction. Reclaimed asphalt pavement (RAP) can be incorporated into new mixtures without significant compromise to performance [172]. Bressi et al. quantified that containing RAP reduces the primary energy demand and the CO_2_-eq. emissions per ton of material in relation to those associated with the reference solution [173]. Recycling asphalt reduces landfill waste and construction costs, contributing significantly to environmental sustainability and circular economy principles [174,175]. Alizadeh et al. [176] confirmed the economic benefits of RAP integration, while Boaventura et al. [177] showed that RAP use does not negatively affect drainage or soil stability.

- Industrial Applications

In industry, asphalt is applied for damp-proofing, protective pipe coatings, soundproofing, and railway track beds [178]. Setiawan et al. found that asphalt track beds enhance vibration isolation, improve drainage, and reduce noise transmission, thereby improving long-term track performance [179]. This demonstrates asphalt’s multifunctional nature in specialised industrial environments.

Performance modifiers significantly expand asphalt’s adaptability. Polymer-modified asphalt (PMA), nano-modified asphalt, bio-asphalt, and rubberized asphalt enhance mechanical durability, thermal stability, and fire resistance [180,181]. Nanomaterials such as nano-clay and nano-silica improve ignition resistance, making asphalt suitable for tunnels and wildfire-prone areas [182,183]. These modifiers ensure asphalt remains competitive in performance-intensive applications [184].

### 4.2. Circular Economy Contributions

Bio-based flame retardants can improve fire resistance and reduce emissions of toxic substances [185]. For example, natural polymers such as lignin, chitosan, and alginate can promote char formation and slow the release of volatile substances [186,187,188,189,190]. Flame-retardant polymers (FRPCs) can also enhance the cohesion, insulation, and mechanical strength of binders [191]. The combination of hydroxyapatite-polydopamine with SBS-modified bitumen can improve interfacial adhesion and fire resistance [192]. Despite these advantages, large-scale application of biomass-derived materials still faces challenges in terms of fire resistance after aging [193,194,195,196].

**Figure 3:**
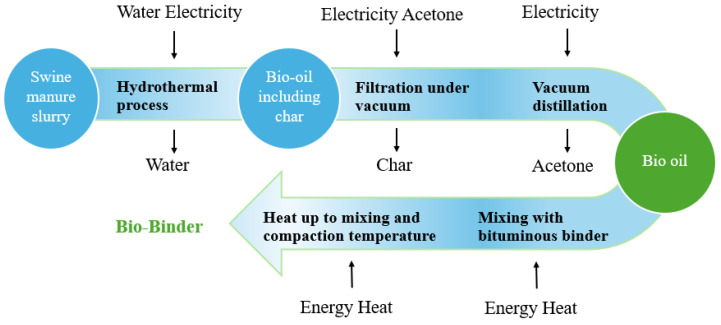
Bio-binder production process chain overview.

In addition to bio-based binders, industrial byproducts are also sustainable aggregate alternatives. Residues from the palm oil industry, including palm oil clinker, fibre, palm kernel shells, and fuel ash, have been successfully incorporated into bitumen mixtures [197,198,199,200]. This dual benefit aligns with circular economy principles, reduces dependence on virgin aggregates and bitumen, lowers greenhouse gas emissions, and improves environmental performance [201,202,203,204]. Figure 3 shows the production process of bio-adhesives, illustrating how biomass feedstocks are converted into bio-oils. In summary, the combination of bio-based adhesives and industrial by-products provides a pathway for developing sustainable refractory bitumen mixtures that balance environmental objectives and economic feasibility [205].

Asphalt has also gained increasing attention for its potential in waste utilization and environmental protection. While its largest application remains in road and highway construction, significant use is also found in building projects, industrial applications, recycling initiatives, and sustainability-oriented retrofits [206]. This distribution reflects both the traditional dominance of asphalt in transportation infra-structure and the growing importance of sustainable, high-performance, and circular economy solutions [207]. In essence, asphalt has evolved from a conventional paving material into a versatile engineering resource capable of meeting diverse mechanical, environmental, and economic demands [208]. These broad and evolving applications further underscore the need for reliable and standardized evaluation methods to ensure that both conventional and modified asphalt, particularly formulations incorporating polymers or flame retardants which can achieve the required performance in varied service environments.

## 5. Evaluation Methodology

The combination of precision manufacturing processes, comprehensive experimental testing, and advanced computational modelling provides a comprehensive framework for developing flame-retardant asphalt mixtures that meet the safety and durability standards for practical infrastructure applications [209]. Post-fire assessments, including residual stiffness and crack resistance, validate that flame-retardant modification does not affect the long-term durability of pavements. Computational simulations such as FEA and thermodynamic models can predict thermal gradients, stress distributions, and degradation kinetics, and capture the interactions between binders, aggregates, and additives. These tools can improve the accuracy of performance predictions by simulating emissions and potential toxic byproducts, guide mixture optimization, and support life cycle and environmental impact assessments [210].

### 5.1. Fire-Performance Assessment Techniques

When evaluating the fire resistance of asphalt, the cone calorimeter method conforming to ASTM E1354 is recommended as the primary evaluation method [26]. This technique reliably and repeatably measures ignition behaviour, exothermic rates, and combustion characteristics under controlled, application-relevant conditions. For a deeper understanding of thermal decomposition mechanisms, microscale techniques such as microscale combustion calorimetry (MCC) and TGA can be used as supplementary methods.

Table 4 summarises the key experimental techniques used to evaluate FR asphalt, each offering different scales, parameters, and insights. No single method can capture the full thermal and fire behaviour of FR asphalt. Cone calorimetry remains the benchmark for ignition and heat release analysis but is often supplemented by micro-scale methods such as MCC and TGA, which provide detailed decomposition information but lack real-world combustion conditions. Coupled tools like FTIR-TGA reveal gas-evolution pathways, though they require specialised interpretation. Rheological tests (e.g., DSR) offer valuable post-fire mechanical data but do not measure combustion directly. Therefore, combining thermal, mechanical, and chemical techniques is essential for a comprehensive assessment of FR asphalt performance.

### 5.2. Fabrication

As agencies shift towards performance-based design approaches, the importance of understanding the fabrication methods of asphalt mixtures has increased significantly. Specimen preparation, including material handling, mixing temperatures, equipment, and compaction techniques, can critically influence the measured performance characteristics of the resulting asphalt mix. In the context of rubberised asphalt, three main fabrication processes are commonly employed to incorporate Crumb Rubber Modifier (CRM) into the asphalt system: the Wet Process, the Dry Process, and the Semi-Wet Process. Each process follows a unique method of integrating rubber into the mix, which significantly affects the resulting material performance [217].

#### 5.2.1. Dry Process (DP)

**Figure 4:**
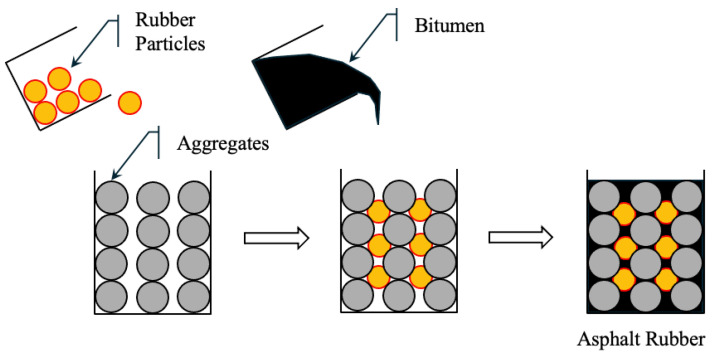
Dry process of asphalt.

In DP, CRM is incorporated directly into pre-heated aggregates prior to the addition of hot binder. Unlike the wet process, the rubber is not pre-reacted with bitumen but is instead dispersed during the final mixing and compaction stages. CRM in this case acts more as a filler or elastic aggregate component than a binder modifier. CRM is first blended with aggregates and then mixed with binder. This method is also illustrated schematically in Figure 4, showing the sequential addition of rubber, aggregates, and bitumen [218]. This approach offers several advantages, including lower equipment requirements and simpler production. It is a cost-effective alternative for incorporating recycled rubber into asphalt mixtures. A notable commercial application of this method is the PlusRide™ system [219]. However, the performance of DP mixes can be inconsistent due to limited interaction between the rubber and binder, potentially affecting long-term durability, fatigue resistance, and overall mechanical performance.

#### 5.2.2. Wet Process (WP)

**Figure 5:**
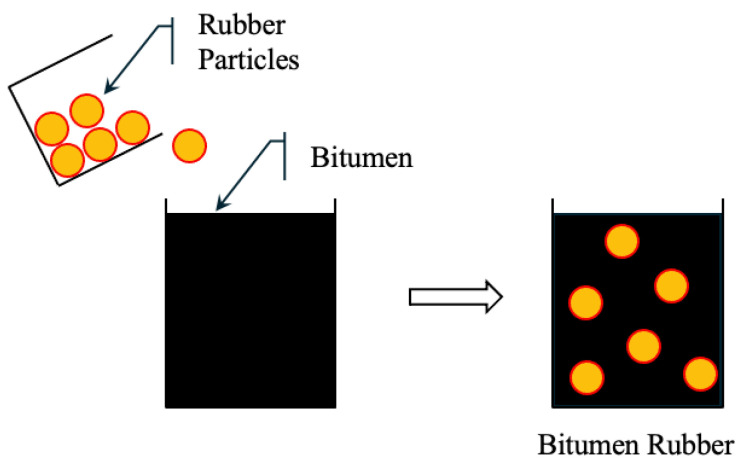
Wet process of asphalt.

In the wet process (WP), finely ground CRM is mixed with hot bitumen binder (typically with a penetration of 60/70) in a high-shear mixer. The rubber undergoes partial digestion and expansion, and sometimes partial depolymerization, resulting in an asphalt–rubber (AR) binder with enhanced mechanical and rheological properties. The AR binder is then mixed with aggregates to form rubberized bitumen. Figure 5 shows rubber particles uniformly dispersed in the binder [218]. WP mixtures offer higher elasticity, rutting resistance, and crack resistance, making them suitable for high-stress applications. However, the process requires specialized equipment and strict temperature and time control, which increases cost and operational complexity.

#### 5.2.3. Semi-Wet Process (SWP)

**Figure 6:**
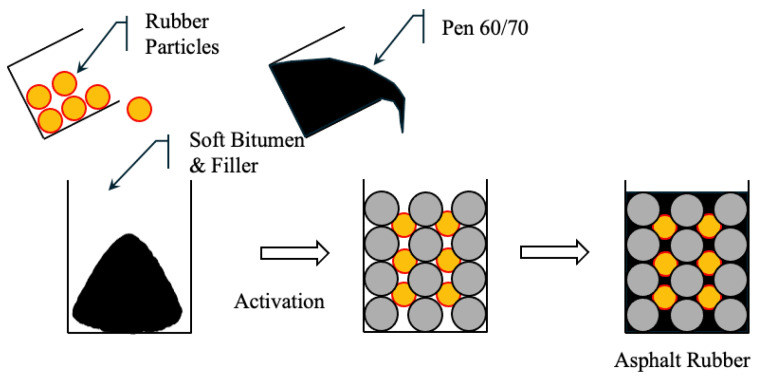
Semi-Wet process of asphalt.

The semi-wet process is a hybrid technique that combines the advantages of wet and dry processes while reducing their limitations. As shown in Figure 6, this method is well-suited for RAP) where mixing efficiency and binder compatibility are critical. In this method, CRM is first mixed with soft asphalt and mineral fillers to produce active rubber particles (ARP). These particles are partially digested and pre-expanded to improve their compatibility and dispersibility in asphalt mixtures. The active rubber particles are mixed with aggregates and then a conventional 60/70 penetration grade binder is added to form a homogeneous rubber-modified asphalt mixture. The production process of active rubber particles involves mixing 60/70 penetration grade asphalt with 20 wt% of aromatic extract (viscosity of 25 mm^2^/s at 100 °C) in a high-speed shear mixer at 1000 rpm for 10 min [217] to produce a soft binder (penetration 115) that enhances the absorption and expansion of the rubber. This creates an oil-free particle surface, thereby improving its interaction with aged and new asphalt. In addition, lime filler (<75 μm) is used to enhance the particle structure. The process requires only some basic equipment, such as a temperature-controlled heating vessel and a standard mixer, so it can be adopted by small asphalt producers [220].

Table 5 summarises the advantages and limitations of each rubber modification process used in asphalt. The WP provides high performance but involves greater cost and operational complexity. The DP is more economical and straightforward, though it may result in inconsistent performance due to limited binder–rubber interaction. The SWP offers a balanced solution, combining good performance with moderate equipment and processing requirements.

SWP produces a dense and cohesive structure, ensuring that the rubber is uniformly dispersed in the asphalt matrix. This structural consistency significantly improves properties such as elasticity, temperature resistance, and crack resistance. It has better compatibility in RAP applications and does not require expensive specialized equipment [219]. Therefore, it is a balanced and efficient solution for producing rubber-modified asphalt.

### 5.3. Characterisation

Understanding the mechanical properties of asphalt mixtures is crucial for evaluating the effectiveness and practical application of flame-retardant materials. While flame-retardant additives primarily improve combustion performance, they also significantly alter the stiffness, tensile strength, resistance to deformation, and high-temperature structural stability of asphalt. These mechanical responses determine the performance of asphalt under thermal stress during fire exposure and influence crack propagation, fatigue resistance, and permanent deformation after heating. Therefore, this section introduces mechanical tests, including tensile testing, fatigue testing, rutting testing, thermal characterization, combustion assessment, and microstructure analysis. These provide key information for a deeper understanding of the interaction between flame-retardant mechanisms and material durability. Integrating these methods allows for a more comprehensive understanding of the performance of asphalt under coupled mechanical and thermodynamic conditions, thereby supporting the design and optimization of next-generation flame-retardant asphalt systems.

#### 5.3.1. Tensile and Compressive Testing

Tensile and compression tests are important methods for evaluating the mechanical properties of asphalt binders and mixtures under stress. They are particularly suitable for studying the performance of materials in application environments where degradation is likely to occur [221,222,223]. By measuring properties such as strength, stiffness, and fracture strain, these tests help determine the conditions under which flame-retardant asphalt begins to fail or lose its functional capacity.

**Figure 7:**
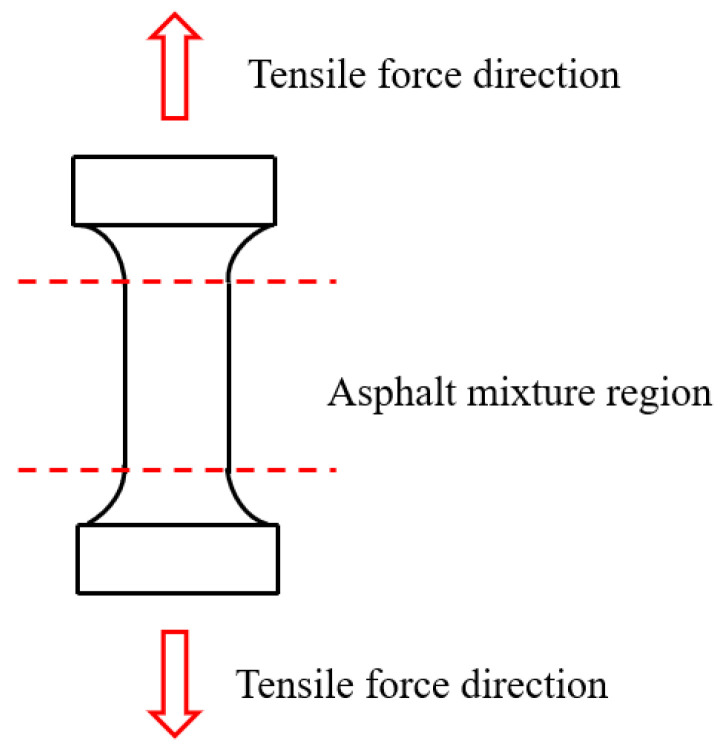
Drectice tensile testing schematic diagram of cylindrical specimen.

Figure 7 presents the tensile testing apparatus for asphalt mortar specimens. Dog-bone shaped specimens are cast in multiple molds to ensure consistent geometry and uniform stress distribution. After curing, the specimens are demolded, trimmed, and their mass and dimensions are checked for uniformity to ensure the repeatability of the test results. During the test, a controlled uniaxial load is applied along the longitudinal axis until fracture, allowing for a quantitative assessment of tensile properties and failure mechanisms [224].

Temperature control during testing ensures consistency with curing conditions, enabling reliable assessment of mechanical behaviour under simulated thermal environments. Tensile testing is particularly important for evaluating cracking resistance and flexibility, as asphalt mixtures generally become more brittle under thermal stress [225].

**Figure 8:**
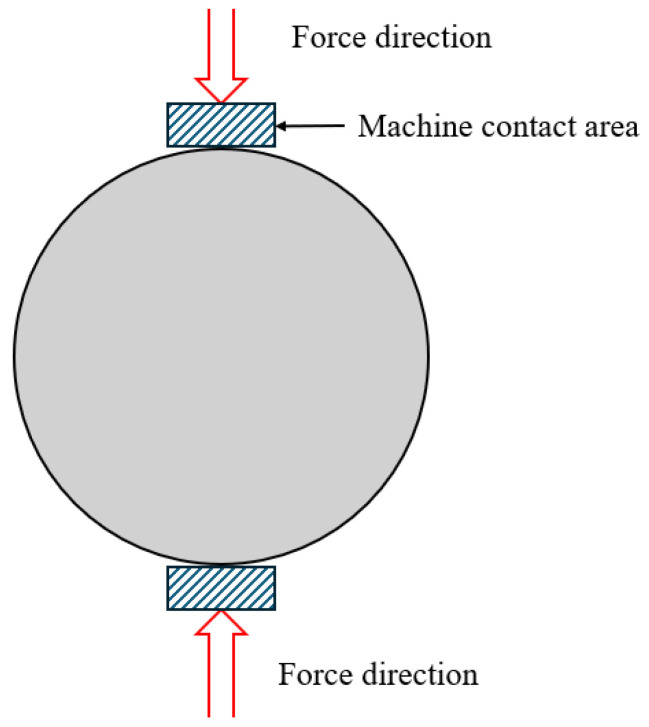
Drectice tensile testing schematic diagram of cylindrical specimen in cross section.

Indirect tensile strength (ITS) testing provides a complementary method for assessing tensile properties under compressive loading. As shown in Figure 8, a compressive load is applied along the diameter of a cylindrical specimen, producing tensile stress perpendicular to the load direction. This test is performed according to EN 12697-23. It assesses the tensile properties induced by compressive loads, providing in-depth insights into crack resistance and structural temperature. Its principle is to assess crack resistance through vertical tensile cracking, thereby indirectly determining tensile strength. The ITS test is simple, reproducible, and suitable for both laboratory and field testing, making it a widely used testing method. In addition to assessing combustion kinetics and thermal decomposition, ITS and compression tests provide a comprehensive evaluation of the fire resistance of bitumen, enabling researchers and engineers to select the most appropriate testing method.

The tensile strength is calculated based on the peak applied load and the specimen’s geometric parameters using Equation (1).

(1) $$\sigma =\frac{2P}{\pi dt}$$

where

- σ = horizontal tensile strength (GPa);
- P = applied load at failure (kN);
- d = specimen diameter (mm);
- t = specimen thickness (mm).

The main advantages of the ITS method include simple specimen preparation, short testing time, low equipment requirements, and low result variability. It can provide in-depth analysis of the bond strength, crack resistance, and fracture behaviour of asphalt mortar under stress. Based on elasticity theory, although the stress distribution is inherently three-dimensional, two-dimensional analysis can effectively simulate the tensile response under specific loads and geometries. Therefore, the comprehensive application of direct tensile strength, indirect tensile strength, and compressive strength tests is the foundation of the performance design method for flame-retardant asphalt systems. This comprehensive testing strategy is particularly important for infrastructure in high-risk environments such as tunnels, airports, and bridge decks [226,227,228].

#### 5.3.2. Fatigue Testing

**Figure 9:**
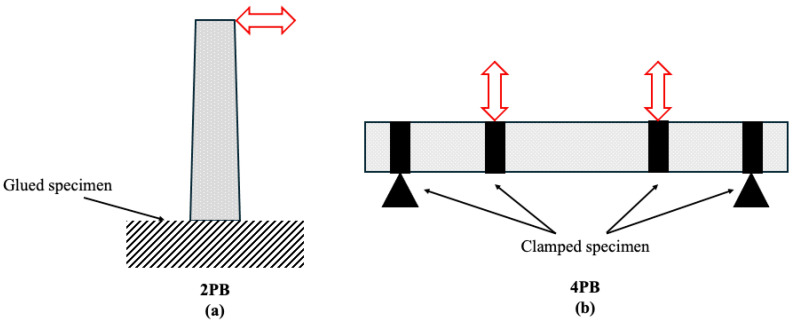
Loading configuration of (**a**) 2PB test and (**b**) 4PB right.

In addition to tensile and compressive strength assessments, fatigue testing is crucial for evaluating the long-term mechanical durability of asphalt mixtures under cyclic loading. Commonly used fatigue testing methods include uniaxial and triaxial compression fatigue tests, as well as the four-point bending beam (4PB) test, the latter being particularly suitable for applications requiring enhanced mechanical toughness [229]. To more realistically capture the conditions of flammable environments, thermomechanical fatigue testing combines high temperatures and cyclic loading. Figure 9 shows the loading configurations and geometric dimensions for both tests, with (Figure 9a) two-point bending (2PB) test and (Figure 9b) four-point bending (4PB) test. These methods reflect the synergistic effect between thermal and mechanical stresses, providing a more comprehensive understanding of how these conditions degrade pavement performance over time [230]. European standards evaluate fatigue using 2PB on trapezoidal and 4PB on prismatic specimens.

Fatigue tests were conducted at various strain amplitudes to obtain a series of fatigue lives (N). Damage curves were generated by plotting the relationship between secant pseudo stiffness (C) and damage parameters for each cycle [231]. During a typical fatigue test on asphalt materials, three distinct phases of stiffness degradation can be observed [229]:

- The rate of stiffness reduction decelerates.
- Stiffness decreases in a nearly linear fashion.
- Stiffness reduction accelerates rapidly.

#### 5.3.3. Wheel-Tracking Test (Pavement Evaluation)

Rutting, or permanent deformation, is a critical performance indicator for asphalt mixtures. Among the available laboratory methods, the wheel-tracking (WT) test is considered one of the most reliable because it closely replicates real traffic conditions. Rutting develops through two primary mechanisms: shear deformation (lateral material movement) and compaction (volume reduction). Shear failure typically occurs within the upper 100 mm of the pavement, especially in hot mix asphalt (HMA) layers, but can penetrate deeper when material support is insufficient. Under repeated loading, rutting forms as a longitudinal depression in the wheel path, often accompanied by lateral heaving [220].

**Figure 10:**
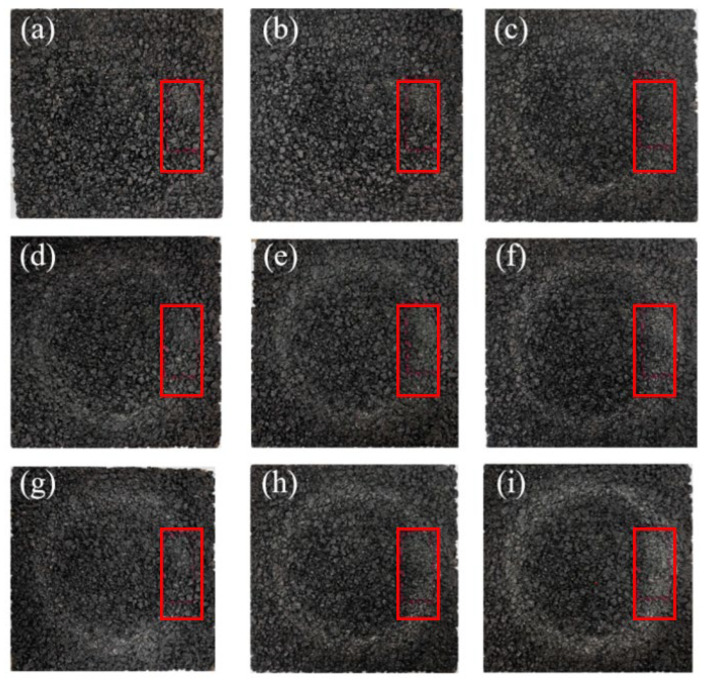
Scanning examples under different abrasion cycles ((**a**–**i**) represent abrasion cycles of 0; 4000; 8000; 12,000; 16,000; 40,000; 80,000; 200,000; and 220,000, respectively). Adapted from [232], MDPI, 2025.

Laser texture scanners were used to measure the macrotexture of the road surface before and after different wear levels, quantifying the texture changes caused by friction tests. Multiple wear cycle stages were scanned within the 80 mm × 40 mm rectangular area shown in the red box in Figure 10, corresponding to the intervals of friction test cycles from 0 to 220k cycles. The mean profile depth (MPD) of the road surface macrotexture is a standard parameter for quantifying the texture related to skid resistance and drainage, where the initial MPD (MPD0) is taken from the unworn area, and the mean profile depth (MPDa) after gradual wear is taken from the scan results of the rut area. The road wear index (PWI) represents the degree of texture degradation, as shown in Equation (2), by converting the ratio of the current MPD to the initial MPD of each surface into a percentage [232]. Higher PWI values indicate better pavement texture retention.

(2) $$PWI=\left(\frac{MPDa}{MPD0}\right)\times 100\%$$

#### 5.3.4. Combustion Behaviour

Cone calorimeter testing is a commonly used standard method for evaluating the flammability and combustion behaviour of materials, including asphalt mixtures. By measuring key parameters such as ignition time, HRR, total heat release (THR), smoke production, and mass loss, this test provides a comprehensive understanding of the combustion performance of asphalt under controlled heat flux conditions (ISO 5660-1) [27,212,233].

**Figure 11:**
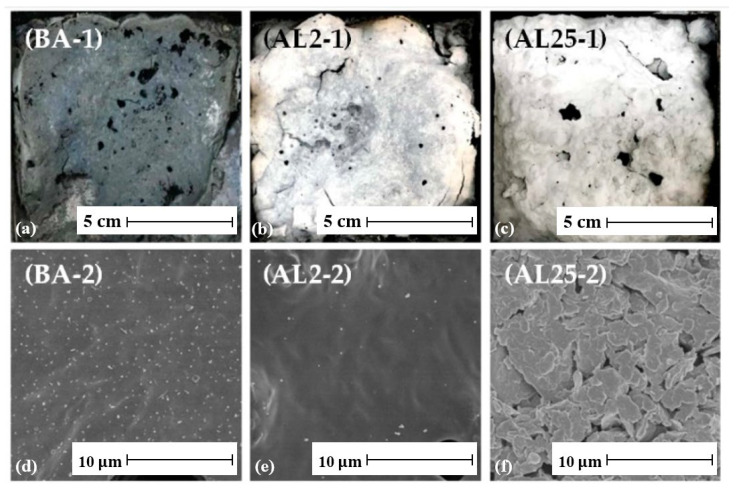
(**a**–**c**) Digital and (**d**–**f**) SEM images of the residues after cone calorimeter tests. Adapted from [234], MDPI, 2019.

This test can also reveal the formation of the barrier layer. Figure 11 shows digital photographs and scanning electron microscope (SEM) images of the residues after the test. The digital photographs (Figure 11a–c) show that AL2 (2 wt.% LDHs) and AL5 (5 wt.% LDHs) retained more structural residues than the base asphalt (BA), indicating better flame retardant properties and a denser, more continuous char layer with fewer surface pores. Scanning electron microscopy results (Figure 11d–f) show that BA forms porous residues due to the release of volatile gases during pyrolysis, while AL2 forms a smoother, crack-free surface, effectively limiting the escape of combustible gases and restricting the transfer of heat and oxygen. The uniform dispersion of LDH also stabilizes the carbon layer. When the LDH content is high, AL25 exhibits a sintered layered morphology, which is due to the structural collapse after LDH decomposition, indicating that the dispersion problem hinders the formation of an effective barrier layer [234].

**Figure 12:**
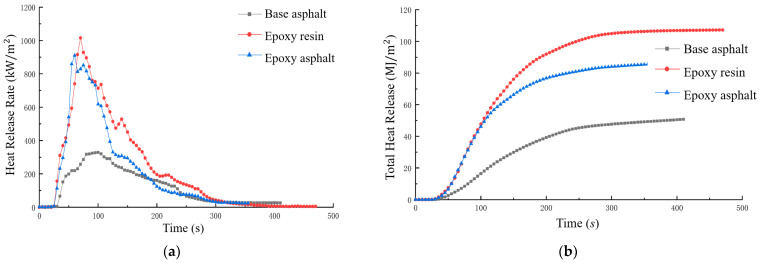
(**a**) HRR and (**b**) THR for comparing asphalt–epoxy samples. Adapted from [236], MDPI, 2022.

The ignition temperature of bitumen is typically between 420 and 450 °C, as determined by cone calorimeter testing [235]. This threshold is influenced by factors such as binder content, aggregate gradation, and flame retardant additives. Additives such as APP and EG have been shown to improve fire resistance by reducing peak HRR and slowing degradation [70,77,79,81]. Figure 12 shows the (Figure 12a) HRR and (Figure 12b) THR of different bituminous epoxy resin samples, highlighting the role of additives in reducing HRR and THR by more than 50% [236].

#### 5.3.5. Thermal Behaviour

Thermal properties are crucial for evaluating the performance and durability of asphalt binders. Thermogravimetric analysis (TGA) is widely used to assess thermal stability and decomposition by tracking mass loss under controlled heating conditions. TGA typically involves heating a small sample (5–10 mg) in a nitrogen atmosphere at a heating rate of 10–20 °C/min to prevent oxidation, followed by heating in an oxygen–nitrogen mixture above 550 °C to analyse combustion. This method allows for a detailed assessment of thermal degradation and oxidative stability [29].

The thermal degradation behavior of CRM particles under a nitrogen atmosphere can be described based on the characteristic patterns observed in TGA and derivative thermogravimetric (DTG) analyses. The mass-loss profile shows three distinct decomposition stages corresponding to the breakdown of different rubber components. The initial decomposition stage occurs at the onset of heating, where a small mass loss is detected as the least stable components begin to volatilize. This is followed by the primary decomposition stage, which represents the most significant mass-loss event. Natural rubber typically undergoes rapid degradation between 300–400 °C, while synthetic rubber decomposes mainly between 400–500 °C [224], producing the main DTG peak associated with the highest rate of mass loss. After this major event, a secondary and slower degradation stage is observed at higher temperatures. This stage reflects the decomposition of carbon-rich residues, fillers, and stabilizers, resulting in a gradual decline in mass until a stable char portion remains.

These characteristic temperature intervals and decomposition features provide valuable indicators of the aging behavior of recycled rubber asphalt. Furthermore, the degradation index estimated from the TGA curve offers a quantitative measure of polymer deterioration within recycled RAP materials [237].

To further characterise thermal degradation mechanisms, TG and DTG curves for epoxy asphalt, revealing three primary degradation stages: (1) resin and hydrocarbon decomposition below 387 °C, (2) cracking and gas evolution from 387–467 °C (peak ≈ 440.8 °C), and (3) combustion of residuals and semi-coke between 467–580 °C (peak ≈ 510.9 °C) [236]. These results indicate that the degradation mechanism of epoxy asphalt is gradual, involving polymer decomposition, volatile release, carbonization, and oxidation. These findings are crucial for the development of flame-retardant asphalt mixtures, enabling targeted use of additives and design adjustments based on thermal properties. However, generalizing laboratory results to real-world fire scenarios and understanding the long-term effects such as additive synergy and material aging remain challenges [238].

#### 5.3.6. Microstructure

Microstructural characterisation of asphalt binders and mixtures is vital for understanding their performance and durability. SEM provides high-resolution imaging of asphalt’s surface morphology, fracture patterns, phase distribution, and adhesion mechanisms at micro- to nanoscale. SEM uses a focused electron beam to generate signals such as secondary electrons and backscattered electrons revealing topographical and compositional details. Modern SEM systems include multiple detectors and often Energy Dispersive X-ray Spectroscopy (EDS) for elemental analysis, though EDS is less effective for volatile organic asphalt components due to beam-induced volatilisation [239].

**Figure 13:**
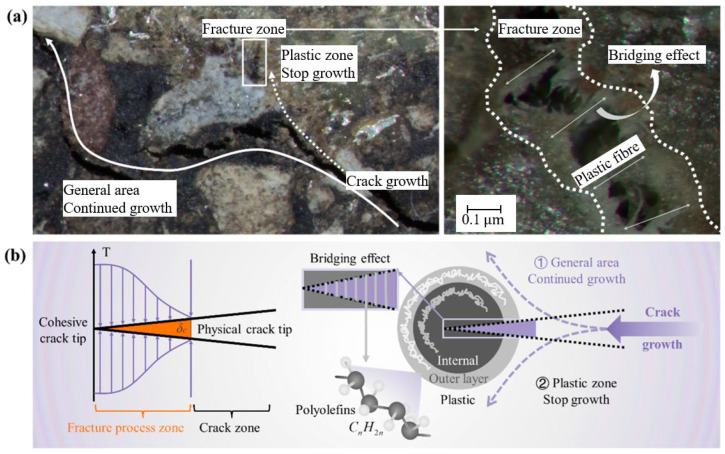
Fracture of asphalt mixture containing plastic: (**a**) fracture diagram; (**b**) fracture theory. Adapted from [240], MDPI, 2025.

The nonlinear fracture process can be described by a cohesive zone model, which includes four distinct stages: (1) the undamaged stage, (2) the crack initiation stage, (3) the material softening stage, and (4) the critical crack formation stage [240]. Scanning electron microscopy revealed the trend of crack propagation, with cracks mainly occurring in the matrix and the plastic zone. The microstructure in Figure 13a shows that the crack propagates along the path of local lowest energy, bypassing the hard second-phase particles. Theoretically, when a crack passes through the plastic zone, the polyolefin molecular chains rearrange and slip, forming plastic fibres that hinder crack propagation. The thermal effects during manufacturing lead to the formation of a bilayer structure in the plastic material, as shown in Figure 13b. Therefore, the presence of the plastic phase in the asphalt mixture effectively reduces crack initiation and alters the crack propagation path, resulting in a reduction in the number of cracks compared to the matrix region [240].

**Figure 14:**
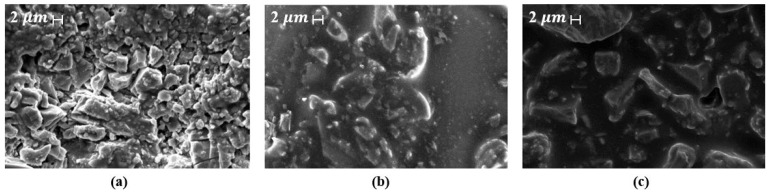
SEM images: (**a**) conventional asphalt mixture with 5.1% bitumen, (**b**) wet mixing asphalt sample with 3.6% bitumen and 1.5% pyrolysis carbon black, (**c**) dry mixing asphalt sample with 3.6% bitumen and 1.5% pyrolysis carbon black. Adapted from [240], ScienceDirect, 2025.

Figure 14 shows SEM micrographs of asphalt samples prepared by different mixing methods: (Figure 14a) conventional asphalt with 5.1% bitumen, and (Figure 14b,c) wet and dry mixes with identical proportions of asphalt, aggregate, and pyrolysis carbon black, revealing differences in volumetric properties [240]. In FR asphalt, SEM analysis before and after fire exposure highlights changes in surface roughness, char formation, and binder degradation, providing insight into thermal protection and FR performance [239,240]. SEM is also essential for examining binder interfaces and microcracks, which influence mechanical stability and aging resistance [241,242].

The synergistic effect of SEM and EDS lies in the elemental mapping of charred residues and binder components, enabling the detection of elements such as phosphorus, magnesium, and silicon originating from flame retardants (e.g., APP, MH, or nano-silica). The microstructure arrangement of polymers affects elasticity, adhesion, and overall fire resistance [58,60,159]. Furthermore, SEM helps assess moisture sensitivity by observing peeling and binder–aggregate debonding after curing treatments such as wet–dry cycles and freeze–thaw cycles. Microstructural evidence provided by SEM helps assess whether additives can improve the adhesion and durability of binders under combined thermal and wet stress. Key SEM parameters for assessing the microstructure of asphalt include surface morphology, char layer thickness, binder coverage uniformity, and the presence of microcracks or voids before and after fire exposure.

### 5.4. Computational Analysis

Computational analysis is increasingly essential for understanding combustion in asphalt mixtures, particularly with FR additives. While conventional tests like cone calorimetry provide valuable data, they are time-consuming and costly. CFD, FEA, and MD simulations offer detailed studies of flame propagation, heat transfer, and thermal decomposition, providing key parameters such as burning velocity, ignition delay, and toxic emissions [32,243].

These models have successfully replicated real-world fire scenarios, including tunnel fires, airport pavements, and bridge decks. FDS simulates smoke and flame spread, evaluating how additives like expandable graphite and ATH delay ignition [6]. ANSYS thermal–structural simulations predict strain gradients in asphalt layers, demonstrating mechanical benefits of LDH [244]. MD simulations reveal molecular-level degradation pathways in binders modified with nano-silica or magnesium hydroxide, showing suppression of volatile release, reduced peak heat release, and potential self-healing effects [245]. Together, these techniques bridge lab experiments and full-scale fire scenarios.

Each method contributes uniquely, FDS captures large-scale fire dynamics, ANSYS models heat transfer and structural response, and MD provides nanoscale insights into additive interactions. However, FDS often oversimplifies asphalt as homogeneous, neglecting multiphase effects, while MD findings are underutilized in macro-scale models, leaving a gap between molecular and system-level predictions [244,245,246].

#### 5.4.1. Fire Dynamic Simulator

**Figure 15:**
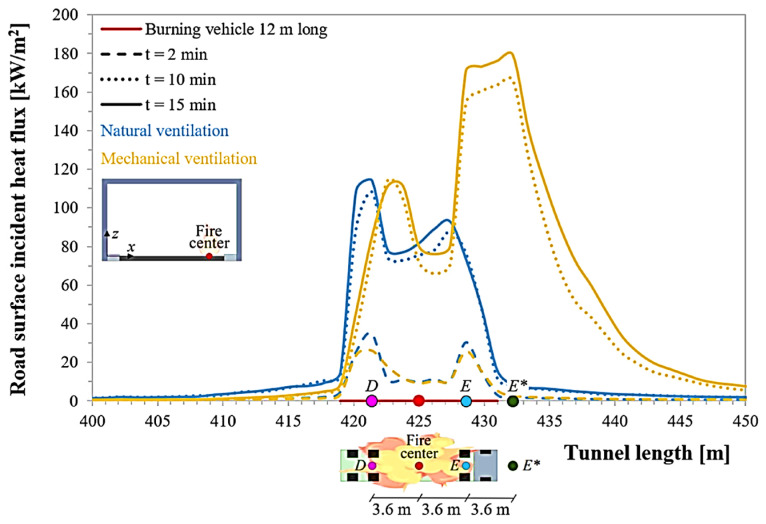
Longitudinal profiles of the incident heat flux on the road surface, with reference to the axis passing through the centre of the burning heavy goods vehicle, after different time (t) from the fire start, considering both the cases of natural and longitudinal mechanical ventilation. In a naturally ventilated tunnel, the peak surface temperature occurs at point D; in a longitudinally ventilated tunnel, the peak surface temperature occurs at point E (or point D). Within 10 min of the fire, when the tunnel is mechanically ventilated, the peak surface temperature occurs at point E* (in other words, due to the activation of the longitudinal mechanical ventilation system, the peak location moves from point E to point E* over time); however, for a naturally ventilated tunnel, the peak surface temperature still occurs at point D. Adapted from [32], MDPI, 2024.

FDS is a powerful CFD-based tool widely used to simulate various fire scenarios, from material-scale combustion to complex tunnel fires. By integrating combustion chemistry, fluid dynamics, and heat transfer, FDS can predict heat flux, smoke movement, and pyrolysis-related phenomena in asphalt systems in detail [27]. In tunnel fire studies, FDS can effectively simulate heat release, smoke stratification, and air velocity to gain insights into the performance of fire-retardant pavements [32]. Figure 15 shows the evolution of heat flux stratification in a tunnel fire scenario. The heat flux increases significantly over time, indicating that smoke accumulates rapidly along the tunnel roof [6]. In the future, developing coupled models that integrate material decomposition and gas-phase combustion remains a key direction. Accurate tunnel geometry, ventilation settings, and vehicle load inputs are also crucial for generating realistic simulation results [17].

#### 5.4.2. ANSYS Modelling

ANSYS analysis is widely used to simulate transient heat transfer and thermodynamic response of asphalt pavements under fire conditions. These models capture temperature distributions between layers and assess the interaction between surface and bulk combustion [82,147]. Advanced combustion models, such as the combustion rate model and the extended coherent flame model, can simulate flame propagation, binder pyrolysis, and gas emissions, while representative volume elements can provide detailed predictions of thermal shielding effects and mechanical behaviour [247,248].

**Figure 16:**
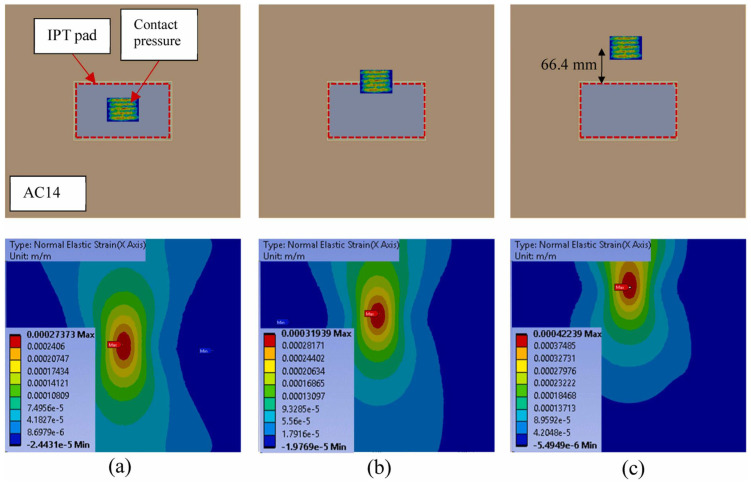
The strain distributions at the bottom of asphalt for the wheel located (**a**) at the centre, (**b**) at the edge of the IPT pad, and (**c**) 66.4 mm from the edge of the pad. Reproduced from [244], ScienceDirect, 2024.

Figure 16 shows that the location of wheel loads significantly affects the strain distribution at the bottom of the asphalt layer. The figure shows that moving the static wheel load from the centre of the pavement to the edge (66.4 mm from the centre) increases the maximum tensile strain by 16%, while moving the load to 66.4 mm from the edge increases the maximum tensile strain by 54%. This is mainly attributed to two reasons: (1) the difference in stiffness (and mass) between the IPT subbase and the surrounding asphalt pavement; and (2) the different distances of the load location from the boundary under different conditions [244].

#### 5.4.3. Molecular Dynamic

Molecular dynamics (MD) simulations provide insights into the thermal and chemical behaviour of asphalt binders at the atomic level, particularly the interactions between SARAs under fire conditions [249]. Lighter components rapidly volatilize and support combustion, while heavier asphaltenes decompose slowly. This phased decomposition forms the basis of asphalt combustion behaviour, where volatile components sustain the flame, while asphaltenes enhance fire resistance by forming a char layer [250,251]. Reactive molecular dynamics (rNEMD) can further predict key properties such as glass transition temperature, diffusion coefficient, and thermal conductivity [252]. MD has been applied to systems with modifiers such as graphene, nano clay, and layered hydroxides, demonstrating that enhanced molecular packing and interfacial adhesion can improve thermal conductivity, oxidation resistance, and flame retardancy [251,252,253].

While molecular dynamics (MD) can link molecular mechanisms to macroscopic fire behaviour, several challenges remain [254]. Many force fields derived from general hydrocarbons may not fully characterize the complex heteroatom and polar chemistry of asphalt. Furthermore, the limited spatial and temporal scales of molecular transformation (MD) restrict its ability to predict long-term changes such as aging, additive migration, and cracking. It needs to be combined with larger-scale models such as computational flow CFD and flame diffusion simulation (FDS) to link molecular transformations with tunnel-scale fire simulations and improve the accuracy of predictions for ignition threshold, char yield, and mechanical stability [255].

## 6. Conclusions and Outlook

This review presents a comprehensive overview of asphalt as a polymer-modified material, highlighting its chemical composition, structural characteristics, performance-enhancing modifications, applications, and evaluation methodologies. The complex molecular architecture of asphalt, combined with its inherent heterogeneity, underpins its unique viscoelastic and mechanical properties. FR additives such as APP, EG, and ATH consistently enhance fire resistance by promoting char formation, endothermic decomposition, and gas dilution, although the exact magnitude of improvement depends on the type of additive, dosage, asphalt composition, and testing conditions. Lignin-modified asphalt, for example, has been shown to increase char yield and delay ignition, illustrating the potential for improved thermal stability and fire performance. Advanced formulations, such as polymer–nanomaterial hybrids and crosslinked systems, further enhance binder stability, adhesion, and durability under extreme environmental and loading conditions. Evaluation techniques, including rheology, thermal analysis, microscopic characterization, and mechanical testing, have elucidated the structure–property relationships essential for performance optimization. Collectively, these insights position polymer-modified asphalt as a high-performance, adaptable, and environmentally conscious material, suitable for modern infrastructure applications.

Future research should prioritize the development of multifunctional and sustainable asphalt formulations. Incorporating bio-based, recycled, and hybrid polymers can reduce environmental impacts while maintaining or enhancing mechanical and thermal performance. Also, further research is needed to optimize polymer–bitumen interactions and improve the dispersion of nanomaterials, as these are key factors affecting long-term durability, thermal stability, and refractoriness. Combining experimental research with computational modelling, molecular simulations, and data-driven methods can accelerate the rational design of asphalt formulations tailored to different climates and traffic conditions. Standardization of evaluation protocols, alongside in situ monitoring, will facilitate the translation of laboratory innovations to large-scale applications. Key challenges remain, including understanding long-term polymer–asphalt interactions, optimizing nanomaterial dispersion, and maximizing the performance of recycled polymers. Addressing these gaps will support the development of next-generation asphalt technologies that balance mechanical resilience, sustainability, and cost-effectiveness, providing a foundation for durable and high-performance infrastructure.

## Figures and Tables

**Table 1:** Components of the asphalt matrix and their impact on mechanical and fire performances.

Matrix Component	Role in Asphalt Matrix	Impact on Mechanical Properties	Impact on Fire Performance	Ref.
**Bituminous Binder**	Continuous phase binding aggregates	Provides cohesion, flexibility, viscoelastic behaviour	Thermal stability influenced by SARA fractions; char formation	[19,41]
**Saturates**	Part of maltene fraction; governs binder flow	Affects viscosity and low-temperature flexibility	Volatile and combustible; contribute to ignition	[19,54,55]
**Aromatics**	Maltene component; adhesion and flow properties	Improves binder ductility and adhesion	Volatile; affects smoke and emissions	[42,56,57]
**Resins**	Intermediate polarity; stabilize asphaltenes	Enhances binder adhesion and stiffness	Influence char and residue formation	[42,43,44,58]
**Asphaltenes**	Dispersed phase providing stiffness	Increases binder stiffness and high-temperature performance	Promote char formation; improve fire resistance	[19,45,47,59]
**Mineral** **Aggregates**	Load-bearing skeleton	Provides strength and stability	Certain aggregates (e.g., limestone) release water vapor via endothermic decomposition, aiding fire retardancy	[47,60]
**Limestone**	Specific aggregate type	Enhances mechanical stability	Releases water vapor; cools and dilutes combustible gases	[48,49]
**Polymer** **Modifiers**	Improve mechanical and thermal properties	Enhances elasticity, fatigue resistance, and rutting resistance	Increase thermal stability; may delay ignition but can increase VOC emissions	[50]
**SBS**	Styrene–butadiene–styrene block copolymer	Improves elasticity and resistance to deformation	Enhances thermal stability; potential VOC emission	[51,52]
**Crumb** **Rubber**	Recycled rubber particles	Increases flexibility and fatigue life	Improves fire resistance; potential environmental concerns	[53]

**Table 2:** Comparison of mineral flame retardants for asphalt additives.

Additives	Decomposition Temperature	Mechanism	Fire Benefits	Drawbacks	Ref.
**EG**	~200–300 °C (expansion)	Rapid expansion forming insulating char layer	Forms physical barrier, reduces heat transfer & smoke, stabilizes binder surface	Requires good dispersion, cost considerations	[76,132]
**APP**	~250–300 °C	Intumescent char formation; acid source for char promotion	Excellent PHRR reduction; promotes insulating, phosphorus-rich char	Moisture sensitivity, binder compatibility issues	[81,98,133]
**ATH**	200–300 °C	Endothermic decomposition, water release	Reduces PHRR, delays ignition, lowers smoke/toxicity	High loading reduces flexibility and toughness	[3,82]
**MH**	~340 °C	Endothermic decomposition, water vapor release, MgO residue	Effective heat absorption; smoke suppression; stable ceramic-like barrier	Brittleness at high loading, dispersion challenges	[87,88,90]
**Calcium Hydroxide**	~400 °C	Water release cooling; neutralizes acidic degradation products	Enhances fire retardancy and aging resistance	Limited data on long-term effects	[92,93,134]
**Layered Double Hydroxides**	>300 °C	Water release + acid gas scavenging	Barrier effect; stable char formation	Dispersion difficulties, higher cost	[135,136,137]
**Bio-based Additives** **(e.g., lignin, cellulose, tannins)**	200–350 °C	Promote char formation, thermal stability via natural polymers	Improve char yield, reduce flammability and smoke, eco-friendly	Compatibility and dispersion challenges	[65,107,138,139]
**Polymer Modifiers** **(e.g., SBS, EVA, EVA-grafted polymers)**	>300 °C (polymer dependent)	Modify binder rheology, promote char cohesion, and thermal resistance	Enhance mechanical durability and fire resistance	Potential thermal degradation, cost, processing complexity	[66,140,141,142]
**Fibres** **(e.g., basalt, glass)**	>400 °C	Physical reinforcement; char layer stabilization	Increases mechanical strength, slows fire spread, enhances char cohesion	Potential dispersion issues, cost	[117,143,144,145]
**Nanoparticles Reinforcement** **(CNTs, nano-clays)**	>300 °C–400 °C	Barrier formation, radical scavenging, char reinforcement	Significantly reduces HRR, smoke; improves char strength and thermal stability	High cost, dispersion and processing challenges	[66,67,146]
**MH + Synergy LDH**	~340 °C & >300 °C	Dual endothermic decomposition + barrier effect	Multi-stage heat absorption; enhanced HRR control	Increased stiffness, dispersion complexity	[136,137,147]

**Table 3:** Summary of key asphalt properties, their measurement methods, and the implications of FR modification.

Property	Standard Test/Measurement	Relevance to Asphalt Performance	Effect of FR Additives	Implications
**Penetration**	Needle penetration at 25 °C	Indicates binder softness and consistency	Can reduce penetration (increasing stiffness)	Improved rutting resistance but risk of brittleness in cold climates
**Softening Point**	Ring & Ball test	Represents temperature where binder softens	Often increased by additives	Higher thermal stability, delayed softening under fire exposure
**Ductility**	Ductility test (elongation before fracture)	Shows flexibility and crack resistance	May decrease due to stiffening effect	Potential loss of flexibility and higher cracking risk
**Viscoelasticity**	Dynamic Shear Rheometer (|G*|, δ)	Balance of elastic vs. viscous behaviour	Nanofillers and IFRS increase elasticity and stiffness	Enhanced rutting resistance, possible reduction in fatigue life
**Thermal Stability**	Thermogravimetric & oxidative aging tests	Determines resistance to thermal degradation and aging	Char-forming additives delay ignition and oxidation	Improved flame resistance and extended service life

**Table 4:** Comparative overview of fire-performance evaluation methods in asphalt research.

Method	Measured Parameters	Advantages	Limitations	Ref.
**Cone Calorimeter Test**	Ignition time, PHRR, THR, smoke production	Comprehensive fire behaviour data; standardized benchmark	Limited to bench-scale; may not represent full-scale fire events	[27]
**TGA**	Mass loss vs. temperature, decomposition onset, char yield	High precision; ideal for screening additives; minimal sample size	Does not replicate flaming combustion; pyrolysis only	[211]
**Smoke Density Chamber Test**	Optical density, smoke growth rate, light transmission loss	Assesses smoke toxicity and visibility, vital for tunnels/confined spaces	Lacks heat release data; limited real fire scenario representation	[212]
**Marshall Stability Test**	Flow value, binder stiffness post-exposure	Quick, simple; widely used mechanical screening method	Not a fire test; indirect evaluation of thermal effects	[213]
**MCC**	Heat Release Capacity (HRC), combustion energy per mass unit	Highly sensitive; minimal sample requirement; ideal for research	Not representative of large-scale fire behaviour	[214]
**FTIR + TGA** **Coupled Analysis**	CO_2_, CO, H_2_O, VOCs evolved during decomposition	Enables decomposition pathway mapping; real-time gas tracking	Complex setup; data requires expert analysis	[29,211]
**Cone Calorimetry + Oxygen** **Consumption**	HRR and oxygen demand integrated with ignition metrics	Combined oxygen and heat data provide enhanced fire risk profiling	Technically complex; requires integrated interpretation	[215]
**DSR Testing**	Complex modulus (G*), phase angle (δ), stiffness recovery post-fire	Assesses rheological performance after fire exposure	Post-event analysis only; not a fire test	[216]

**Table 5:** Summary of asphalt fabrication methods.

Process	Rubber Interaction	Equipment Needed	Mixing Complexity	Performance	Cost
WP	High (binder is chemically modified)	High-shear mixers	Complex	Excellent	High
DP	Low (rubber used as filler)	Standard mixers	Simple	Moderate	Low
SWP	Medium (pre-activated rubber)	Basic heating and mixing	Moderate	High	Medium

## Data Availability

The original contributions presented in the study are included in the article, further inquiries can be directed to the corresponding author.

## Abbreviations

The following abbreviations are used in this manuscript:

ARP	Activated Rubber Pellets
ATH	Aluminium Hydroxide
APP	Ammonium Polyphosphate
AR	Asphalt Rubber
BA	Base Asphalt
CO_2_	Carbon Dioxide
CO	Carbon Monoxide
CNTs	Carbon Nanotubes
CFD	Computational Fluid Dynamics
CRM	Crumb Rubber Modifier
DTG	Derivative Thermogravimetric
DP	Dey Process
DSC	Differential Scanning Calorimetry
DSR	Dynamic Shear Rheometry
EDS	Energy Dispersive X-ray Spectroscopy
EGA	Evolved Gas Analysis
EG	Expandable Graphite
FRPCs	Fibre-Reinforced Polymer Composites
FEA	Finite Element Analysis
FDS	Fire Dynamics Simulator
FR	Fire Retardant
FTIR	Fourier Transform Infrared
HRR	Heat Release Rates
HMA	Hot Mix Asphalt
IFS	Indirect Tensile Strength
IFRs	Intumescent Flame Retardants
LDH	Layered Double Hydroxides
LCA	Life Cycle Assessment
MH	Magnesium Hydroxide
MCC	Micro-scale Combustion Calorimeter
MD	Molecular Dynamics
MPD	Mean Profile Depth
PAH	Polycyclic Aromatic Hydrocarbons
PAV	Pressure Aging Vessel
PMA	Polymer-Modified Asphalt
PWI	Pavement Wear Index
RAP	Reclaimed Asphalt Pavement
SARA	Saturates, Aromatics, Resins, and Asphaltenes
SEM	Scanning Electron Microscopy
SWP	Semi-Wet Process
SBS	Styrene–Butadiene–Styrene
TGA	Thermogravimetric Analysis
THR	Total Heat Release
UTW	Ultra-Thin White
VOCs	Volatile Organic Compounds
WMA	Warm-Mix Asphalt
WP	Wet Process
WT	Wheel-Tracking
